# Efficient transfection of Atlantic salmon primary hepatocyte cells for functional assays and gene editing

**DOI:** 10.1093/g3journal/jkad039

**Published:** 2023-02-14

**Authors:** Alex K Datsomor, Ragnhild Wilberg, Jacob S Torgersen, Simen R Sandve, Thomas N Harvey

**Affiliations:** Department of Animal and Aquacultural Sciences, Faculty of Biosciences, Centre for Integrative Genetics (CIGENE), Norwegian University of Life Sciences, Ås, 1433, Norway; Department of Animal and Aquacultural Sciences, Faculty of Biosciences, Centre for Integrative Genetics (CIGENE), Norwegian University of Life Sciences, Ås, 1433, Norway; AquaGen AS, P. O. 1240, Trondheim, 7462, Norway; Department of Animal and Aquacultural Sciences, Faculty of Biosciences, Centre for Integrative Genetics (CIGENE), Norwegian University of Life Sciences, Ås, 1433, Norway; Department of Animal and Aquacultural Sciences, Faculty of Biosciences, Centre for Integrative Genetics (CIGENE), Norwegian University of Life Sciences, Ås, 1433, Norway

**Keywords:** Atlantic salmon, primary cells, cell culture, CRISPR, hepatocyte

## Abstract

The expansion of genomic resources for Atlantic salmon over the past half decade has enabled efficient interrogation of genetic traits by large-scale correlation of genotype to phenotype. Moving from correlation to causation will require genotype–phenotype relationships to be tested experimentally in a cost-efficient and cell context-relevant manner. To enable such future experiments, we have developed a method for the isolation and genetic manipulation of primary hepatocytes from Atlantic salmon for use in heterologous expression, reporter assay, and gene editing experiments. We chose the liver as the tissue of interest because it is the metabolic hub and many current Atlantic salmon research projects focus on understanding metabolic processes to improve traits such as the growth rate, total fat content, and omega-3 content. We find that isolated primary hepatocytes are optimally transfected with both plasmid and ribonucleoprotein using a Neon electroporator at 1,400 V, 10 ms, and 2 pulses. Transfection efficiency with plasmid and cutting efficiency with ribonucleoprotein were optimally 46% and 60%, respectively. We also demonstrate a 26 times increase in luciferase expression under the promoter of the key liver metabolic gene, *elovl5b*, compared to an empty vector, in line with expected liver-specific expression. Taken together, this work provides a valuable resource enabling transfection and gene editing experiments in a context-relevant and cost-effective system.

## Introduction

The release of a high-quality reference genome for Atlantic salmon in 2016 (Lien *et al.* 2016) enabled efficient scans for genome-wide genotype–phenotype associations. This resulted in more efficient breeding programs through marker-assisted and genomic selection (Houston *et al.* 2020) and a step change in our ability to understand the genetics of traits in domestic and wild populations (Barson *et al.* 2015). Yet, the majority of genotype–trait associations are a result of a linkage disequilibrium to unknown causative variants, and this limits the utility of such associations in wild population management and breeding (Daetwyler *et al.* 2013).

Moving forward, development of genomic resources and tools to help tease apart correlation from causation will be of great importance for applied and basic research on the genetics of Atlantic salmon traits. One such initiative is Functional Annotation of Animal Genomes (FAANG) (Andersson *et al.* 2015; Clark *et al.* 2020), which aims to systematically generate and archive functional genomics phenotypes such as gene expression, chromatin accessibility, and histone tail modifications across tissues and developmental stages and cell types. This data can then be used to provide genome regulatory context of genetic variants associated with phenotypes, which takes us one step closer to causal relationships. However, to fully bridge the genotype–phenotype gap and dissect out gene function and causal variants using reporter assays or CRISPR-based approaches, efficient protocols for transfection of DNA or proteins into cells are needed.

Several studies have demonstrated transfection of Atlantic salmon cell lines using electroporation of DNA and CRISPR components (Schiøtz *et al.* 2011; Gratacap *et al.* 2020), with reports of transfection efficiency ranging between 10% and 90% (Schiøtz *et al.* 2011). Even though continuous cell lines can be excellent systems to study many aspects of cell biology and genetics, the immortalization process and lab evolution often lead to cells with different properties than their tissue of origin (Lopes-Ramos *et al.* 2017; Ben-David *et al.* 2018). Hence, for certain applications, primary cell cultures are preferred over continuous cell lines, as they often bear stronger functional resemblance to the cells in vivo (Zeilinger *et al.* 2016; Nagarajan *et al.* 2019). Unfortunately, for Atlantic salmon primary cells, efficient transfection protocols are lacking. Standard chemical transfection has proven extremely inefficient in primary cells of Atlantic salmon and other teleosts. For example, chemical transfection of primary gill and liver cells from rainbow trout and Atlantic salmon, respectively, failed to reach 1% transfection efficiency (Romoren *et al.* 2005; Wilberg 2020). Furthermore, only a few cell lines from Atlantic salmon are in fact available for the research community and none from key metabolic tissues like the liver. There is therefore a pressing need to develop efficient transfection protocols which will enable functional genomics in primary cells, as well as aid in developing new Atlantic salmon cell lines. The current study describes an efficient transfection protocol for Atlantic salmon primary hepatocytes and employs the optimal protocol for functional assays and CRISPR/Cas9-based studies.

## Materials and methods

### Isolation of Atlantic salmon primary hepatocytes

Atlantic salmon (*Salmo salar*) parrs of 100–400 g were obtained from the Centre for Fish Research, NMBU. Fish were euthanized by a sharp blow to the head, and the liver immediately perfused for 10 minutes with ice-cold wash buffer, pH = 7.4 (1× Hank's Balanced Salt Solution (HBSS), without Mg^2+^/Ca^2+^, 1 mM EDTA, 10 mM HEPES) via the portal vein. The liver was subsequently perfused for 10 minutes with ice-cold collagenase buffer, pH = 7.5 (1× HBSS with Mg^2+^/Ca^2+^, 10 mM HEPES, 150 U/ml collagenase) via the portal vein, gently torn into small pieces, and incubated for 1 hour in a sterile Erlenmeyer flask with collagenase buffer at 15°C under atmospheric conditions with continuous slow stirring on a magnetic stirrer. Dissociated hepatocytes were thereafter filtered through a 100 µM cell strainer and rinsed with ice-cold Leibovitz's L-15 medium with GlutaMAX supplement (ThermoFisher Scientific). Hepatocytes were harvested at 100× g for 5 minutes at 4°C, resuspended in 5 ml 1× HBSS (without Mg^2+^/Ca^2+^) and spun down again at 100× g at 4°C for 5 mins. Then, the cells were resuspended in 5 ml 1× HBSS (without Mg^2+^/Ca^2+^) and counted using the hemocytometer with trypan blue. Polyethylenimine-coated plates were used to facilitate attachment of hepatocytes in all experiments. Protocol has been published on protocols.io (http://dx.doi.org/10.17504/protocols.io.j8nlkw4p1l5r/v1).

### Optimization of the electroporation-based transfection protocol

Electroporation of salmon primary hepatocytes was performed using the Neon Transfection System (Invitrogen) in accordance with the manufacturer's protocol. Approximately 4 × 10^5^ hepatocytes were transfected with 3 µg of reporter plasmid, pEGFP-N1-FLAG (Addgene# 60360) per well of a 6-well plate. Transfection was performed using electroporation programs with varying voltage, pulse width, and pulse number (Table 1). Transfected cells were incubated in L15 medium (L15 GlutaMAX, 5% fetal bovine serum, without antibiotics) at 15°C under atmospheric conditions. 24 hours after transfection, media was replaced with fresh complete L15 medium (L15 GlutaMAX, 5% fetal bovine serum, 1× streptomycin–penicillin) and cells were incubated at 15°C under atmospheric conditions for an additional 24 hours. At 48 hours post-transfection, the impact of the various programs on cell viability was assessed by resazurin viability assay (Sigma) in accordance with the manufacturer's protocol. Successful transfection was evaluated by GFP expression using the ZEISS fluorescence microscope and the proportion of transfected cells was determined by flow cytometry using the Amnis CellStream (Luminex).

**Table 1.** Neon electroporator parameters for the four programs tested.

Program ID	Voltage (V)	Pulse width (ms)	Pulse number
P2	1400	20	1
P9	1400	30	1
P16	1400	20	2
P20	1150	30	2

### Implementation of the optimal transfection protocol for comparative promoter study

To demonstrate the importance of successful transfection and its potential for functional studies in primary hepatocytes, we employed the optimal transfection program in a luciferase assay for the Atlantic salmon *elovl5b* gene that has shown a liver-specific expression pattern (Morais *et al.* 2009).

### Preparation of promoter luciferase construct

We generated a promoter luciferase reporter construct for *elovl5b* using the pGL4.10[luc2] vector (Promega, GenBank accession# AY738222), which contains the firefly luciferase reporter gene. This vector includes the wild-type “full-length” promoter (pGL4.10-elovl5bWT) which contains the *elovl5b* promoter region NC_027327.1: 27244001–27245560 (Supplementary File 1). This vector is hereafter termed as elovl5bWT. The promoter was amplified from Atlantic salmon genomic DNA using the Platinum SuperFi PCR Master Mix (ThermoFisher) with primers containing 15 bp tails homologous to cloning sites within the pGL4.10[luc2] vector which is necessary for cloning using the In-Fusion HD Cloning Kit (TaKaRa). Primer sequences are indicated in Supplementary Table 1. The promoter reporter vector was isolated using the ZymoPURE plasmid miniprep kit and confirmed by Sanger sequencing (LightRun Tube Sequencing Service, Eurofins).

### Transfection of promoter luciferase constructs and luciferase assay

Approximately 1.0–1.5 × 10^5^ isolated primary hepatocytes were co-transfected per well in 24-well plates with 1.5 µg of promoter reporter construct and 0.5 µg of the reference reporter construct, pGL4.75[hRluc/CMV] (Promega), encoding Renilla luciferase. Transfection was performed using electroporation program P16 (Table 1). At 24 hours post-transfection, fresh complete L15 medium (L15 GlutaMAX, 5% fetal bovine serum, 1× streptomycin–penicillin) was added to transfected cells and incubated at 15°C under atmospheric conditions for an additional 24 hours. To quantify firefly and Renilla luciferase activities, medium on cells was replaced with 100 µl of Dulbecco's modified Eagle's medium (Sigma) and 100 µl Dual-Glo Luciferase reagent (Promega) per well and incubated for approximately 30 minutes. Luminescence was read on a Synergy H1 Hybrid multi-mode microplate reader (BioTek). Luminescence from Renilla luciferase activities was measured 10 minutes after adding 100 µl of Dual-Glo Stop & Glo reagent. Firefly luminescence was normalized to Renilla luciferase luminescence.

### Implementation of the optimal transfection protocol for genome editing using RNPs

To identify the optimal program for genome editing using ribonucleoproteins (RNPs), we designed a guide RNA to Atlantic salmon tp53 (NCBI geneID: 106602901) and performed RNP electroporation using the same programs in Table 1 as described above.

### Preparation and electroporation of RNPs

RNP complexes were prepared according to the Alt-R CRISPR-Cas9 system protocol from IDT. In brief, crRNA:tracrRNA duplexes were made by diluting 2.2 µl of Ssal_tp53_crRNA (200 µM) and 2.2 µl of tracrRNA (200 µM) in 5.6 µl of IDTE buffer. Cas9 protein was prepared by mixing 12 µl of Alt-R Cas9 protein (62 µM) with 8 µl of buffer R (Invitrogen). gRNA duplexes and Cas9 were then mixed gently and incubated at room temperature for 10 minutes. 1 µl of prepared RNPs was added to 9 µl of cells resuspended in buffer R just before electroporation. After electroporation, cells were distributed directly into wells of a 24-well plate containing 1 ml of culture medium (L15, 5% FBS) without antibiotics. After 24 hours at 15°C, media were replaced with culture medium containing antibiotics and antimycotics (L15, 5% FBS, 100 U/ml Pen-Strep, 2.5 µg/ml amphotericin B) and placed at 15°C. Cells were harvested 72 hours later by washing twice with PBS (pH 7.2) and incubating with 100 µl 0.25% trypsin/EDTA (Invitrogen) until cells detached. 400 µl of culture medium containing FBS was then added to inactivate the trypsin. Cells were centrifuged at 150× g for 5 minutes and washed twice in PBS and then stored at −20°C.

### Determination of CRISPR editing efficiency

Genomic DNA was extracted from frozen cell pellets according to the QIAGEN blood and tissue kit protocol. Genomic loci containing the Cas9 cut site were amplified by PCR using the primers Ssal_tp53_seq_fwd and Ssal_tp53_seq_rev (Table 2). PCR reactions were set up as follows: 25 µl Platinum II Green Hot Start PCR Master Mix (Invitrogen), 1 µl each primer (10 µM), 7.5 µl gDNA (∼10 ng/µl), and 15.5 µl nuclease-free H_2_O. Thermocycler conditions were as follows: initial denaturation of 94°C for 2 minutes followed by 35 cycles of 94°C denaturation for 15 seconds, 60°C annealing for 15 seconds, and 68°C extension for 15 seconds. A single clear band at 844 bp was obtained for all reactions. PCR reactions were cleaned up using a QIAquick PCR Purification Kit (QIAGEN) and Sanger sequencing of PCR products using Ssal_tp53_seq_rev was performed by Eurofins Genomics. Cas9 cutting efficiency was determined by ICE deconvolution (Conant *et al.* 2022) of Sanger sequencing traces using default parameters.

**Table 2.** tp53 targeting crRNA sequence and primer sequences for PCR and sequencing of targeted locus.

Name	Sequence
Ssal_tp53_crRNA	GCTCGTACCACTGCCCCAGG
Ssal_tp53_seq_fwd	AGAGTCAGACAAGAACAATGGG
Ssal_tp53_seq_rev	CTGTCTCAGAGTGTTACCATCC

### Statistical analysis

The effects of different programs on transfection efficiency and viability were determined by one-way analysis of variance (ANOVA), followed by Tukey's multiple comparison test with a *P*-value cutoff of 0.05. Comparison of luminescence from different promoters was assessed using Student's *t*-test and a *P*-value cutoff of 0.05. Results shown are representative of 3 independent experiments. All statistical analyses were performed in R version 4.2.1. (Team 2020) using RStudio (Team 2019).

## Results

### Isolation of primary hepatocytes

**Fig. 1.**
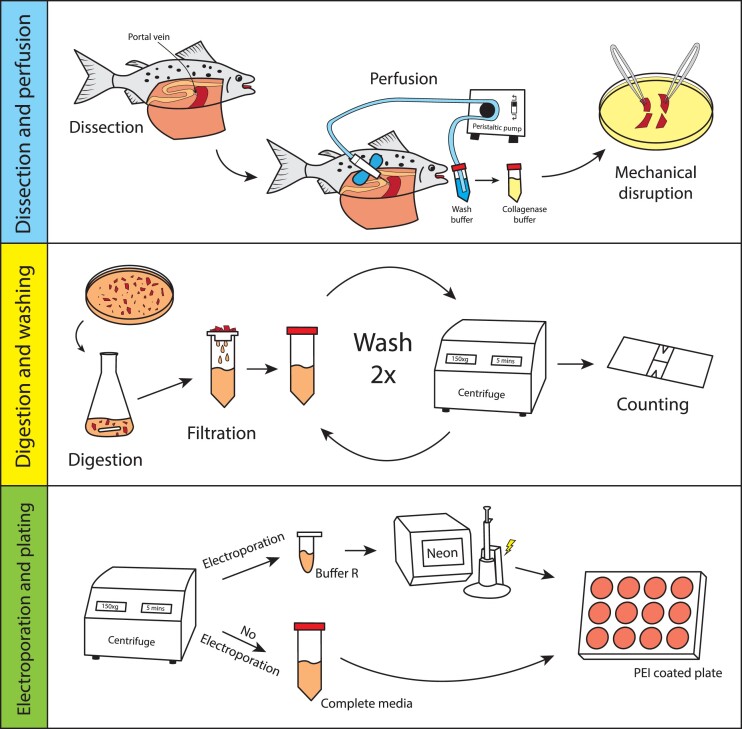
Schematic of the primary hepatocyte isolation procedure and electroporation using the Neon electroporation system.

We isolated primary hepatocytes from freshwater-stage Atlantic salmon (100–400 g) by perfusing the liver in situ through the portal vein with wash buffer to remove erythrocytes, followed by collagenase to digest the extracellular matrix, and several filtration and washing steps to obtain a suspension of single cells (Fig. 1). The isolated primary hepatocytes grew optimally at 15°C under atmospheric condition in L15 medium supplemented with 5% FBS. We found that a liver from a 200 g fish would typically yield between 2 × 10^7^ and 4 × 10^7^ cells with a viability of 80–95% prior to electroporation, as determined by trypan blue staining and counting with a hemocytometer. The isolated primary hepatocytes were mostly dispersed single cells and spherical in shape. We also observed some oval-shaped erythrocytes; however, these were greatly reduced by in situ perfusion. Isolated hepatocytes did not attach to ordinary non-coated culture plates, but when growth surfaces were coated with polyethylenimine (branched), the cells attached optimally at a density of 7–10 × 10^4^ cells/cm^2^. 24 to 48 hours after attachment, cells formed flattened aggregates that would slowly expand. Cells remained viable under our conditions for at least three weeks.

### Transfection of GFP expression plasmid and functional evaluation of the *elovl5b* promoter

**Fig. 2.**
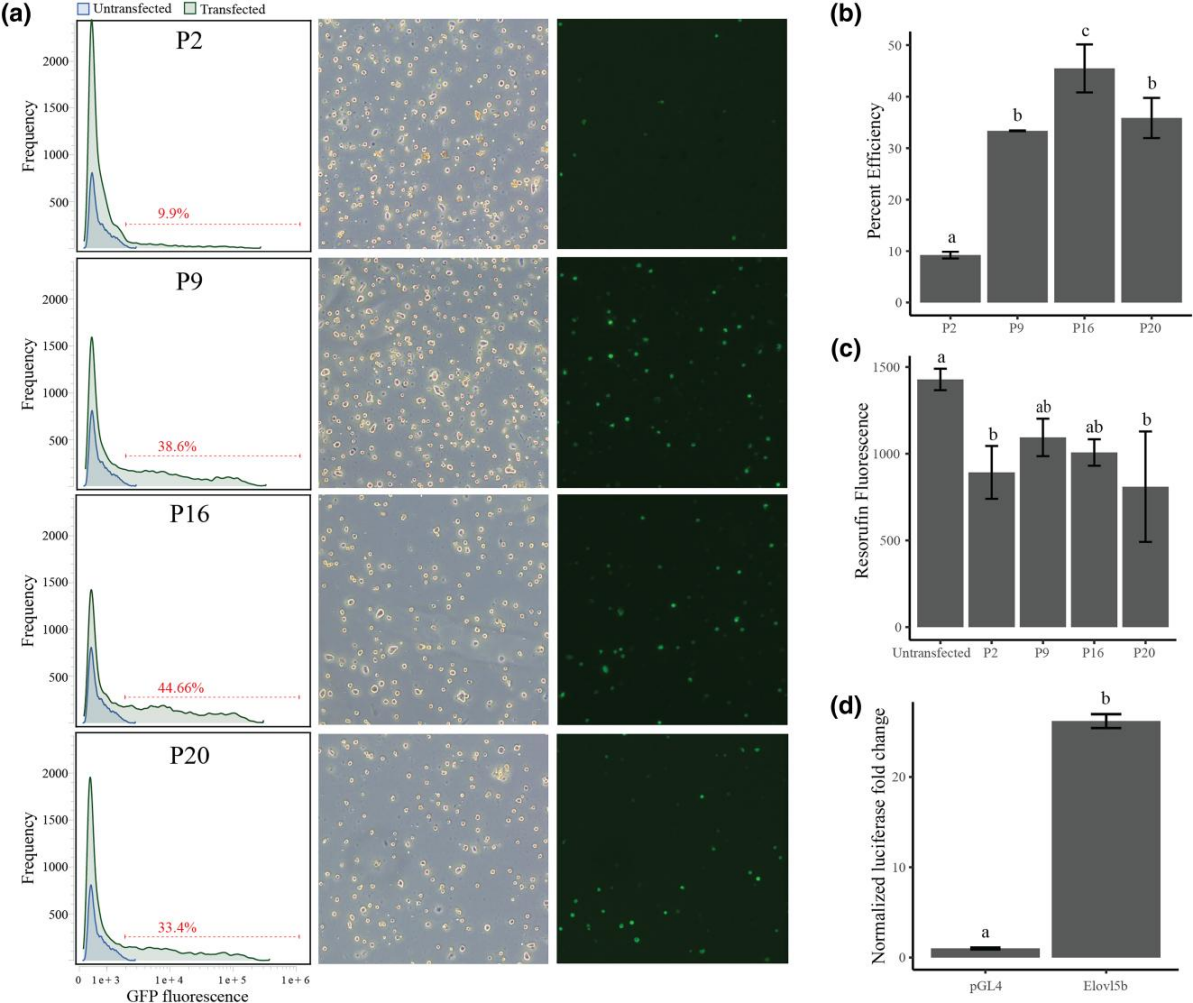
Impact of electroporation programs on transfection efficiency and viability and demonstration of functional reporter assay in Atlantic salmon primary hepatocytes. a and b) The different electroporation programs assessed show varying effects on transfection of hepatocytes with program P16 (Table 1) giving highest transfection efficiency. Transfection efficiency was measured by flow cytometry. c) Evaluation of cell viability by resazurin viability assay at 48-hr post-transfection showed slight reduction in cell viability for all programs compared to non-transfected control. d) Normalized luciferase signal for Atlantic salmon *elovl5b* promoter compared to the empty vector.

We tested 24 electroporation conditions using pGL4.75 which encodes for Renilla luciferase under the control of the CMV promoter. We found a positive correlation between voltage and transfection efficiency, with 1,400 V, 2 pulses having the highest efficiency (Supplementary Fig. 1). Of these conditions, we selected four, three high efficiency and one low efficiency, for further analysis by transfecting with a plasmid encoding GFP under the control of the CMV promoter. Transfection efficiency was measured by fluorescent microscopy and flow cytometry. We found P16 (1400 V, 20 ms, 2 pulses) to have the highest transfection efficiency of 46% (Fig. 2a and b). P2, P9, and P20 had electroporation efficiencies of 9%, 33%, and 36%, respectively. Cell viability, as measured by the conversion of resazurin to resorufin, decreased after electroporation for all conditions with no clear differences between the four conditions (Fig. 2c).

To showcase the utility of the transfection protocol for primary liver cells, we performed a luciferase promoter–reporter assay using the promoter of a known liver centric gene involved in the fatty acid metabolism (*elovl5b*). The *elovl5b* promoter showed significant (*P* < 0.05) 26 times increase in the luciferase signal compared to the empty vector (Fig. 2d).

### CRISPR Cas9 gene editing

**Fig. 3.**
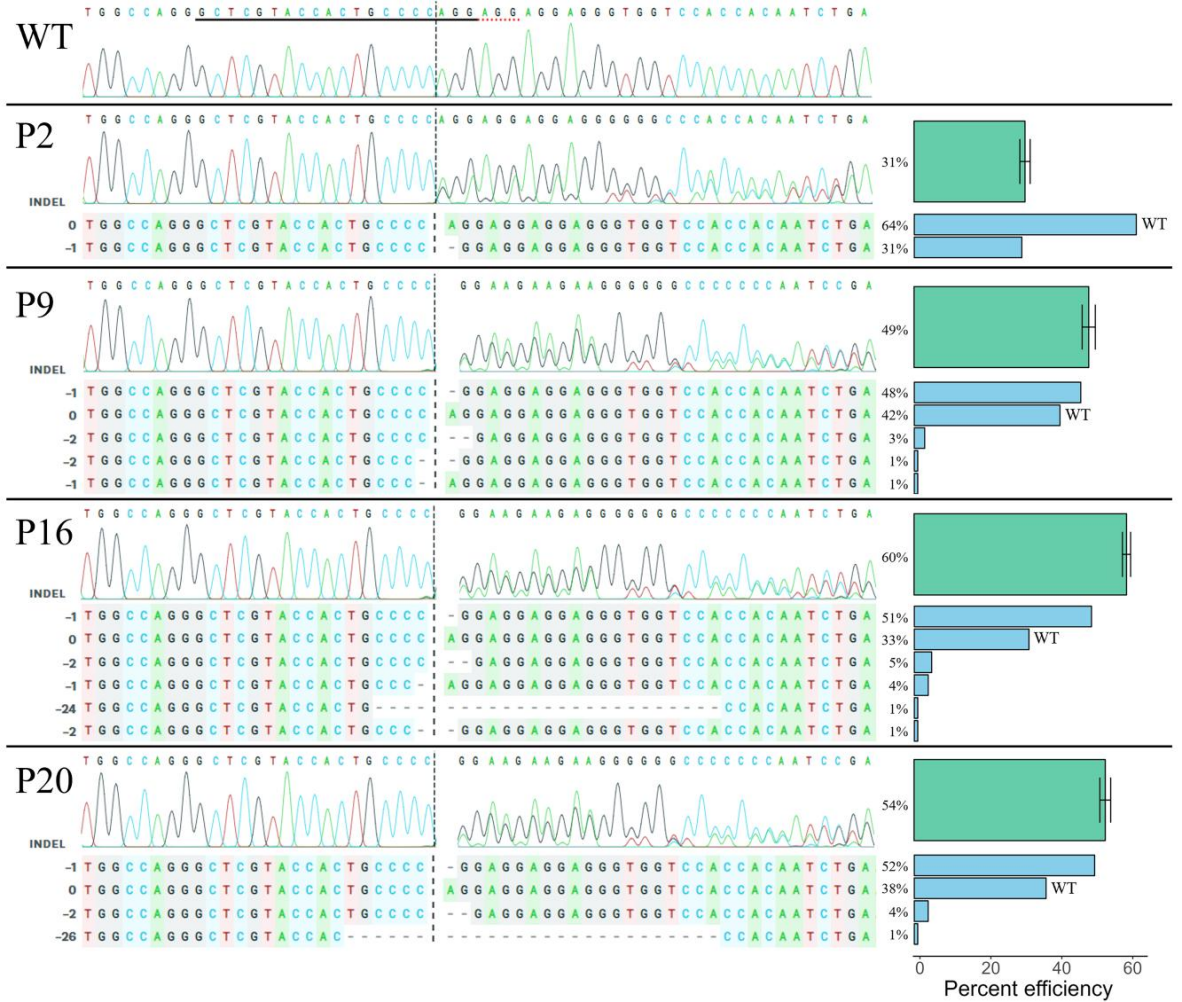
Cutting efficiency of RNPs targeting Atlantic salmon P53 (NCBI geneID:106602901) using four electroporation programs. Sanger sequencing traces for each electroporation program are shown. The gRNA binding site is underlined in the WT trace and the cut site is indicated by a vertical dashed line in all traces. ICE deconvolutions are shown for each program. Bar plots (right) indicate total percent cutting efficiency (wide bars) and contribution of each indel (narrow bars). Indels of length 0 are unaltered WT sequence and negative indels are deletions. No insertions were detected.

To test the effectiveness of gene editing by RNP electroporation in Atlantic salmon primary cells, we designed a single-guide RNA (sgRNA) to one of the three salmon P53 genes (NCBI geneID:106602901). We then combined this with Cas9 protein to form RNPs and electroporated using the four conditions described above. We found that P16 had the highest cutting efficiency of 60%, P9 and P20 had slightly lower efficiencies of 49% and 54%, respectively, and P2 had the lowest of 31% (Fig. 3). ICE deconvolution of Sanger sequencing traces showed that the majority of indels were one or two base pair deletions and none were insertions (Fig. 3).

## Discussion

High-quality in vitro experimental cell model systems for commercially important aquaculture species like Atlantic salmon are very important for implementation of modern molecular techniques. The establishment of high-throughput and robust methods at the cutting edge of molecular biology is necessary for advanced research into genetic mechanisms underlying physiological processes. However, no robust protocols for transfection and genetic engineering of primary cells exist for Atlantic salmon. To this end, we have established an efficient transfection protocol for Atlantic salmon primary hepatocytes, the hub of fat and energy metabolism, and demonstrate our ability to employ these cells for functional and CRISPR/Cas9-based studies.

Transfection efficiency of continuous cell lines and primary cells is highly dependent on the cell type and the method used. In our study, we measured electroporation efficiency for both plasmid DNA and RNPs and found optimally 46% and 60% efficiencies, respectively. Electroporation of Atlantic salmon TO cells and Atlantic salmon kidney (ASK) cells has achieved a plasmid transfection efficiency up to 90% and 50%, respectively (Schiøtz *et al.* 2011). Neon electroporation has been shown to efficiently deliver RNP complexes to salmon head kidney (SHK-1) and ASK cells achieving editing efficiencies as high as 90% (Gratacap *et al.* 2020). This higher efficiency in cell lines compared to our cells is expected because primary cells are notably more difficult to transfect than cell lines.

To our knowledge, our study represents the first protocol of transfection of primary hepatocytes in Atlantic salmon; however, many studies have been conducted in mammalian systems. Human umbilical vein endothelial cells have been demonstrated to achieve plasmid electroporation efficiencies of up to 90% with viability greater than 70% (Gresch and Altrogge 2012). Primary hepatocytes typically have lower electroporation efficiencies between 25% and 40% for plasmid (Chen *et al.* 2005; Gao *et al.* 2012) and 52% and 78% for RNPs (Rathbone *et al.* 2022) which is more in line with our observations in Atlantic salmon hepatocytes. Interestingly, Chen *et al.* (2005) were able to double electroporation efficiency in primary mouse hepatocytes by electroporating cells 24 hours after partial hepatectomy (Chen *et al.* 2005). This demonstrates that the cell growth rate is likely a major factor limiting electroporation efficiencies in primary hepatocytes, so increasing the growth rate through optimization of growth conditions could be a route to improving transfection efficiency.

High-efficiency transfection of primary hepatocytes opens opportunities for modern high-throughput molecular techniques in Atlantic salmon within a physiologically relevant context, for example, massively parallel reporter assays which would enable systematic genome-wide identification of cis-regulatory elements (Wang *et al.* 2018; Kircher *et al.* 2019; Klein *et al.* 2020). The transfection efficiency that we obtained in our study is in the order of what is required for these assays as it enables manageable cell numbers and ensures cost-efficient experimental design. In addition, the promoter of *elovl5b* showed a 26 times increase in activity in the primary hepatocytes consistent with the liver-specific expression pattern of salmon *elovl5b* (Morais *et al.* 2009), which underscores the physiological semblance between isolated hepatocytes and the liver. Our high cutting efficiency of RNP electroporation in primary hepatocytes will enable metabolically relevant *ex vivo* gene knockout studies in Atlantic salmon. For example, recent studies have knocked out key lipid metabolism genes in Atlantic salmon to study the function in vivo (Datsomor *et al.* 2019a, 2019b); however, these fish trials are extremely time-consuming and costly. *Ex vivo* gene editing of primary hepatocytes would enable quicker turnaround times and allow for the elucidation of a wider range of gene functions. Taken together, our protocol for efficient plasmid transfection and gene editing in primary hepatocytes will open a wide variety of opportunities to study hepatic function in Atlantic salmon.

## Supplementary Material

jkad039_Supplementary_Data

## Data Availability

Strains and plasmids are available upon request. The authors affirm that all data required to confirm the conclusions of the article are presented within the article, figures, and tables. Supplemental material available at G3 online.
