# Supplementary material for: Efficient transfection of Atlantic salmon primary hepatocyte cells for functional assays and gene editing
Source: G3 (Bethesda). 2023 Feb 14;13(4):jkad039. doi: 10.1093/g3journal/jkad039 (PMC10085798; doi:10.1093/g3journal/jkad039)
Supplement: jkad039_Supplementary_Data [file jkad039_supplementary_data.zip › Figure_S1_G3-2022-403943.docx]

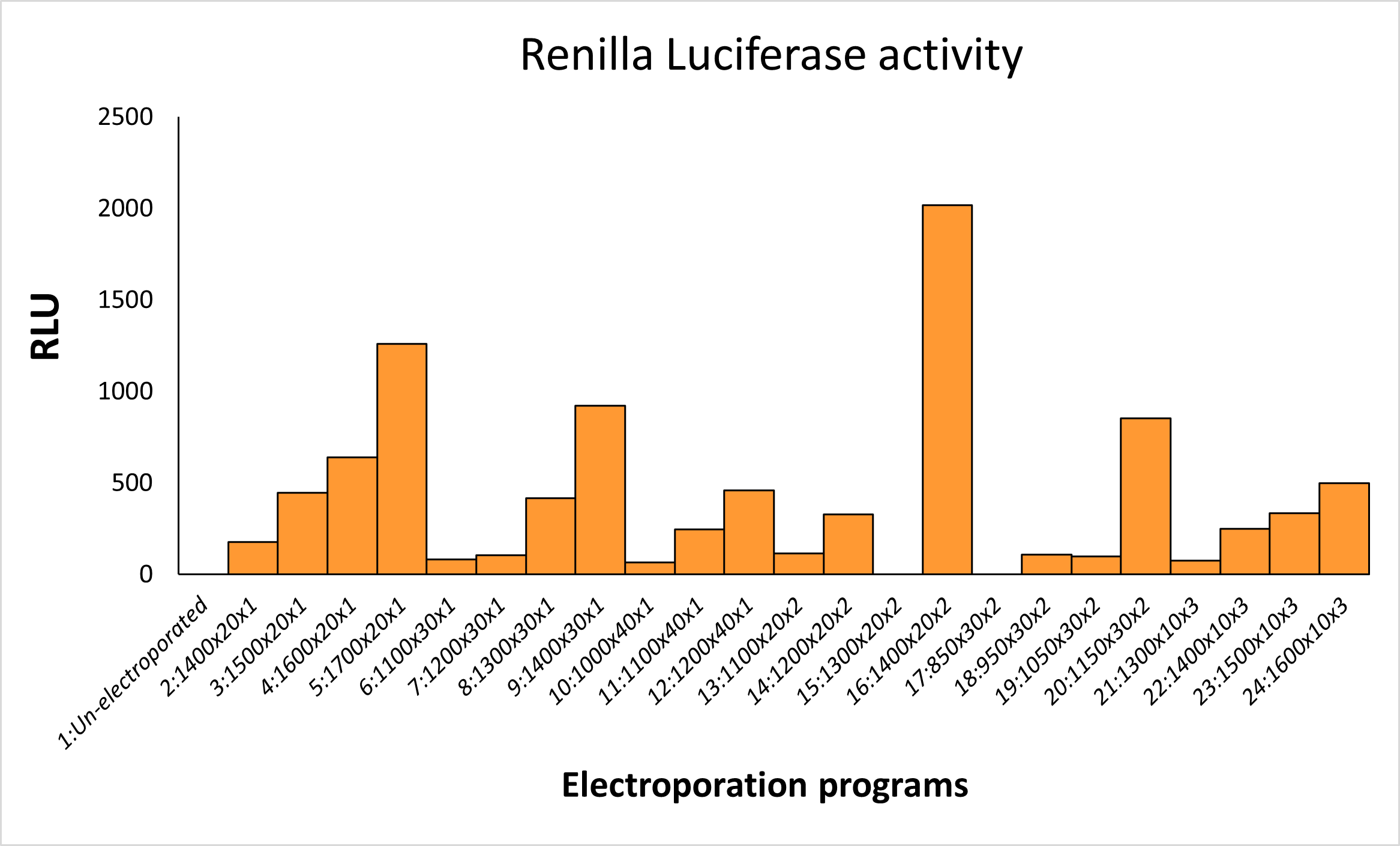


**Figure S1:** Renilla luciferase activity in cells electroporated with 24 different electroporation programs. The Renilla signal is measured in relative light units (RLU). Background signal from un-transfected sample (program 1) is subtracted.
