# Supplementary material for: Efficient transfection of Atlantic salmon primary hepatocyte cells for functional assays and gene editing
Source: G3 (Bethesda). 2023 Feb 14;13(4):jkad039. doi: 10.1093/g3journal/jkad039 (PMC10085798; doi:10.1093/g3journal/jkad039)
Supplement: jkad039_Supplementary_Data [file jkad039_supplementary_data.zip › File_S1_G3-2022-403943.docx]

**File S1:** The Atlantic salmon *elovl5b* promoter region (NC_027327.1: 27244001 – 27245560) cloned into pGL4.10-elovl5bWT.

5’GAGAATGAGGTTAAGGTTAGCAAAAGAGTTAGGGTTAGGCCAAACTAAAAAGCTAAGGTTGTCAACAACTCTTATCCCCGACGCGACAACTAGATGGCGGTGTACTAGTATCCACAACCGCACAGACTATCCCTAAAAAATGCACCAGTATCATAACACAGGTTACTGAGCTCTGCTCTGTGTAGCCATGTGAGTGATGCAGTGATTTGATTGGTCTCCACTGACGAGAAATGAATGGGGTGACATCGGCTTGCTAACTGATTTATTAACTGGTTGTGAGTGATGCAGTGATTTGATTGGTCTCCACTGATGCGAAATGAATGGGGTGACATCGGCTTGCTTACCGACTTGCTATCTGGGACGCATTCAGCAGTGTTGAAGGTTTTGGAACGTTCAGATAGAAATATGCTAAAATATAGCTCAAACATGCCTCTCTGGCATATATAAGGAATCACATCGGCTCTAGTCATGGTATTTCTATCTGCAACGTTCGAGAACGTTTGGCAACTGAACATGGCCTAGGTTGCAGTTATACACGTACCGCGTTCAACCAGTTAGTATGTCGGAAATTCCCGTGAACGCAGCATTGGGCACGTTTTCTTGTCACTTTCTCTAAATCCAGCATGTCACCTATACTATAGGCTACGAATAAAACCTCACGAGTAACCTTGTCCCGACTACAACGCACATCCCGGGTGTCTGTAGCCCAGGCAAAATGTAAGAGTGCACAAATATTATTAAGCACATTGGCTAAAAGAGAATAGCTCTTTGGTATACGTAAATGCGCAGTTGTGCATTTAAAACGTGCCTCTACAACTAAGATTTTCACTACTCAGCAAACCATGCAGTTTATTAGGCTACAGATTAAATAATGCTAAACTTTACAGGGTGGAGACACTGCACGGTGATGAGTTTGATGCTCATTTCCAATACATGATGATCAATGCTTGACCGCTCCATAATAATCTCATCATGTAGCTACCCGCACAGCCTACAAGCACTGTAGCTGTTGGCTAGAGCGCACATGACAAGACCAGAGTAGACTGGCACATTTGCTATTTAATGCAAGAGATTTTGTCACAAAACAATCGGTAGAGTTGAAAATGCTATAAACACATTGAATTTAAGAGGAACGGTACATGTTGTGTGCACTATGTCATCACGCACTGATTTTTATTTGCAACAAGTCGGTTTGGTGGAAAAACAATTGGTAGGAAAATGCCCATATTTTCTTTATGCCGGTTTTAAAATATTCACATGAAAATCTGTCGCCAATTGAATGGAAACCTAGTTTAAGTGGGACGCCAACATCAGCAGACGGTCAAATCACCTTCTGGGTGATGATCACTGCCAATGAGGTAGGCCATTAGGGAGGCTGATAGCCAATTATCTACCATCTGAAGATGATAGCCTAATAGCTAGATCTCGTCACTTATGACGCTCTGGACAATTTGCCATATAGACTGAGTTCATGTGTTTCTCCCGCTGTTTCCACTGACCGACGAGGCTGCACATTTGTGCTTTGGGACCTGGGCAGGCAAGATTACGCATCCTCCAGAG3’
