# Supplementary material for: Efficient transfection of Atlantic salmon primary hepatocyte cells for functional assays and gene editing
Source: G3 (Bethesda). 2023 Feb 14;13(4):jkad039. doi: 10.1093/g3journal/jkad039 (PMC10085798; doi:10.1093/g3journal/jkad039)
Supplement: jkad039_Supplementary_Data [file jkad039_supplementary_data.zip › Table_S1_G3-2022-403943.docx]

**Table S1:** Primers used for cloning pGL4.10-elovl5bWT.

| **Primer name** | **Sequence (5'-3')** |
| --- | --- |
| El5bWTPromFw | ACTGGCCGGTACCTGGAGAATGAGGTTAAGGTTAGCAA |
| El5bWTPromRv | CCGGATTGCCAAGCTCTCTGGAGGATGCGTAATCTTGC |

*****Underlined primer sequences are 15 bp homology arms required for InFusion HD cloning.
